# A Systematic Review of the Effects of EEG Neurofeedback on Patients with Schizophrenia

**DOI:** 10.3390/jpm14070763

**Published:** 2024-07-18

**Authors:** Dan Cătălin Oprea, Iasmin Mawas, Cătălina Andreea Moroșan, Vlad Teodor Iacob, Eliza Mihaela Cămănaru, Ana Caterina Cristofor, Romeo Petru Dobrin, Bogdan Gireadă, Florin Dumitru Petrariu, Roxana Chiriță

**Affiliations:** 1 Department of Medicine III, Faculty of Medicine, Grigore T. Popa University of Medicine and Pharmacy of Iasi, 700115 Iasi, Romania; oprea.dan-catalin@d.umfiasi.ro (D.C.O.); mawas.iasmin@gmail.com (I.M.); andreea.morosan@umfiasi.ro (C.A.M.); vlad-teodor.iacob@umfiasi.ro (V.T.I.); camanaru_eliza-mihaela@d.umfiasi.ro (E.M.C.); caterina.cristofor@umfiasi.ro (A.C.C.); gireada_bogdan@d.umfiasi.ro (B.G.); roxana.chirita@umfiasi.ro (R.C.); 2 Institute of Psychiatry “Socola”, 36 Bucium Street, 700282 Iasi, Romania; 3 Department of Preventive Medicine and Interdisciplinarity, Grigore T. Popa University of Medicine and Pharmacy of Iasi, 700115 Iasi, Romania; florin.petrariu@umfiasi.ro

**Keywords:** electroencephalography neurofeedback, brainwave biofeedback, neurofeedback, schizophrenia, self-regulation, neuromodulation

## Abstract

Schizophrenia is a neuropsychiatric disorder affecting approximately 1 in 300 people worldwide. It is characterized by a range of symptoms, including positive symptoms (delusions, hallucinations, and formal thought disorganization), negative symptoms (anhedonia, alogia, avolition, asociality, and blunted affect), and cognitive impairments (impaired memory, attention, executive function, and processing speed). Current treatments, such as psychopharmacology and psychotherapy, often do not fully address these symptoms, leading to impaired everyday functionality. In recent years, there has been a growing interest in neuromodulation due to computer and engineering science making extraordinary computational advances. Those put together have reinitiated the spark in the field of neurofeedback (NF) as a means for self-regulation and neuromodulation with the potential to alleviate the daily burden of schizophrenia. We review, in a systematic way, the primary reports of electroencephalogram (EEG)-based NF as a therapeutical tool for schizophrenia. The main body of research consists mostly of case studies and case reports. The results of a few randomized controlled studies, combined with case studies/series, underscore the potential use of NF as an add-on treatment option for improving the lives of suffering individuals, being sustained by the changes in brain function and symptomatology improvement. We aim to provide important evidence of neuromodulation using NF in patients with schizophrenia, summarizing the effects and conclusions found in several clinical trials.

## 1. Introduction

Schizophrenia affects 1 in 300 people worldwide, approximately 0.32% of the global population [1], and is among the top 10 global causes of disability [2]. Epidemiological data are often controversial due to the different assessment methods and approaches to data collection in various studies.

Schizophrenia is characterized by distortion of thinking and perception with inappropriate or blunted affects [3]. The symptoms are usually divided into positive (seen as a “gain of function”, such as delusion, hallucination, and formal thought disorganization), negative (seen as a “loss of function”, such as anhedonia, alogia, avolition, asociality, and blunted affect), and cognitive (impaired memory, attention, executive function, and speed of processing) [2].

The degree of functional disruption provoked by this syndrome varies from severe disability to a high level of functionality [4]. A better psychosocial function alongside a better premorbid adjustment is observed in females. Compared to males, they show fewer negative symptoms, especially those linked to expressiveness rather than amotivation [5]. Cognitive impairment is less well defined; some evidence suggests that male schizophrenia patients have greater memory deficits compared to females. Performances in language, visuospatial, and attention domains appear roughly identical [6].

Neurological changes and symptoms of schizophrenia have a strong relationship, being an important field of interest in the latest studies. Co-occurrence of positive symptoms and poor impulse control followed by aggressiveness have a neurobiological basis. Reduction of total cortical volume, global white matter integrity, and additional reduction in dorsolateral prefrontal cortex (DLPC) volume, alongside inferior parietal lobule volume and internal capsule integrity are associated with increased severity of positive symptoms and a decrease in impulse control [7]. 

Negative symptoms appear to be associated with hypofrontality as a neurobiological substrate, more specifically with the cortical thinning of the medial orbitofrontal cortex [8] and the hypoactivation of the ventrolateral prefrontal cortex and ventral striatum [9].

Cognitive symptoms have a significant impact on functioning, memory, speed of processing, and function of execution being an important target for therapy [10]. Abnormal decreased functional connectivity between the cerebellum, frontal, temporal, occipital, and thalamic areas was observed in association with cognitive function [11]. 

Approximately 30% of patients are unresponsive to antipsychotic medication, with a persistence of positive symptoms such as auditory and visual hallucinations. Although clozapine is the treatment choice for psychopharmacological therapy of resistant forms, up to 60% of patients respond poorly to it [12]. Therefore, alternative solutions to manage treatment-resistant cases are mandatory.

Neurofeedback (NF) is an emergent possible treatment option creating a brain–machine interface that provides real-time feedback to the patient during therapy. NF uses the Pavlovian operant conditioning; patients receive real-time feedback, directly perceiving specific neuronal events by using a visual or auditory representation of their own brain activity while performing a cognitive task with the aim of modulating the brain waves/rhythms in the area of interest and thus of being able to optimize cognitive strategies to achieve neuromodulation [13]. In summary, NF brings the subconscious processes into the conscious field using various stimuli, thus allowing the individual to control it. This therapy can be based on different neuronal activity-collecting methods, such as electroencephalogram (EEG), functional magnetic resonance imaging (fMRI), functional near-infrared spectroscopy (fNIRS), and hemoencephalography (HEG). 

Electrodes are placed on the scalp of the patient to collect the electrical activity generated by the brain, which is recorded, processed, and represented in a visual or/and auditory representation in the form of a metaphor (a car race/a song). Positive feedback is given in the form of a ‘’reward’’ (the car is going faster/a song is played right) when the subject maintains the brain waves in the prespecified parameters. With each successive training session, the parameters are made incrementally harder [14,15]. The duration of a session is between 20 and 25 min after the equipment is attached [16].

FMRI neurofeedback, in contrast with EEG-based neurofeedback, measures the blood-oxygen-level-dependent (BOLD) signal, thus reflecting the neuronal activities in an indirect way [17]. Also, as in EEG NF, the signal is represented by using audio/visual stimuli to display the activities in the area of interest, thus enabling voluntary control. The downside is represented by high cost, the fact that the patient needs to stay in a confined space (the scanner), and low temporal resolution [18].

fNIRS NF measures the metabolic activities of the neurons using the difference in the concentration of oxygen in the hemoglobin measured by optical sensors placed on the scalp. The spatial and temporal resolution lies between fMRI- and EEG-based NF, providing higher spatial resolution than EEG and higher temporal resolution than fMRI, the depth being limited to the neocortex [19].

Due to the cost, portability, practical issue, and invasive character, the EEG NF is the most preferred method [20]. This technique has various applications in the field of neuropsychiatric disorders such as depression [21], anxiety [22], Alzheimer’s disease [23], posttraumatic stress disorder [24], attention-deficit hyperactivity disorder [25], sleep disorder [26], eating disorders [27], etc.

These findings sustain the potential use of NF as an ad-on therapy for treatment-resistant symptoms of schizophrenia. We conducted a systematic study of the literature with the purpose of identifying useful and resourceful protocols that can help clinicians and future researchers.

## 2. Materials and Methods

The preferred reporting items for systematic review and meta-analysis (PRISMA) statement guidelines was used to conduct the literature search [28], and we registered the systematic review protocol on the Open Science Framework.

### 2.1. Search Strategy

Relevant studies were identified up to 20 of April 2024 using four databases: PubMed, Web of Science, Scopus, and Embase. The search strategies used for the interrogation of databases was as follows:

PubMed: ((EEG Neurofeedback) OR (Brainwave Biofeedback) OR (EEG Feedback) OR (Electroencephalography Biofeedback) OR (Brainwave Feedback)) AND ((Schizophrenia) OR (Schizophrenic Disorder) OR (Dementia Praecox));

Embase: (“eeg neurofeedback” OR “brainwave biofeedback”/exp OR “brainwave biofeedback” OR “eeg feedback”/exp OR “eeg feedback” OR “electroencephalography biofeedback”/exp OR “electroencephalography biofeedback” OR “brainwave feedback”) AND (“schizophrenia”/exp OR “schizophrenia” OR “schizophrenic disorder” OR “dementia praecox”/exp OR “dementia praecox”);

Scopus: TITLE-ABS-KEY ((“EEG Neurofeedback”) OR (“Brainwave Biofeedback”) OR (“EEG Feedback”) OR (“Electroencephalography Biofeedback”) OR (“Brainwave Feedback”)) AND TITLE-ABS-KEY ((“Schizophrenia”) OR (“Schizophrenic Disorder”) OR (“Dementia Praecox”));

Web of Science: ALL = ((EEG Neurofeedback) OR (Brainwave Biofeedback) OR (EEG Feedback) OR (Electroencephalography Biofeedback) OR (Brainwave Feedback)) AND ALL = ((Schizophrenia) OR (Schizophrenic Disorder) OR (Dementia Praecox)).

### 2.2. Inclusion and Exclusion Criteria

#### 2.2.1. Population

Clinical studies involving adults with schizophrenia were included. The studies that involved other disorders or only healthy individuals were excluded.

#### 2.2.2. Intervention

Neurofeedback was defined as a technique that provides real-time feedback to the individual regarding their own brain activity using visual or auditory representation with the aim of modulating the brain waves/rhythms in the area of inters, thus achieving neuromodulation.

#### 2.2.3. Comparator

The comparison of the studied population is made with healthy subjects and those with schizophrenia without intervention or control. 

#### 2.2.4. Outcome

Studies have to report variation of neurophysiological changes or improvement of symptoms to be included in this study.

#### 2.2.5. Study Design

Only primary research studies were included, represented by randomized controlled trials and non-randomized controlled trials. Because of the scarcity of primary sources, we also decided to include case series and case reports. Reviews, editorial reports, and conference abstracts were excluded.

### 2.3. Selection Process

The studies identified by the search query, adapted for each database, were first screened using the title and the abstract. No language restriction has been used. Studies were included or excluded based on the criteria defined above. The studies included by abstract were rescreened via a reading the full text. Two reviewers, ODC and IM, independently assessed the interrogation results. Disagreements regarding the inclusion/exclusion of an article were settled through discussion and debate with the whole team.

Relevant data were extracted and synthetized in the standardized data table included in this review.

## 3. Results

**Figure 1:**
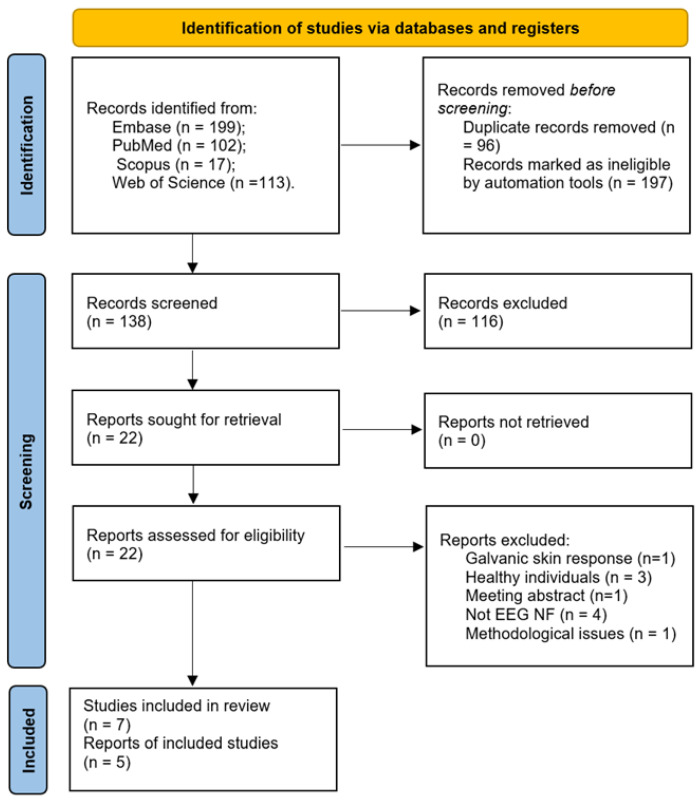
PRISMA flowchart.

Figure 1 summarizes the selection process of the studies screened at each stage. A total of 431 studies were identified from database interrogation. From these, 293 were excluded by automation tools as duplicates or non-clinical trials (conference abstracts, letter to the editor, and editorials). Further, from 138 records automatically screened, 116 were excluded based on the title and the abstract, resulting in 22 eligible papers. Nine more papers were rejected after full text assessment, with the reasons listed in the figure below. Finally, seven studies and five reports were included in this review.

### 3.1. Studies

#### 3.1.1. Randomized Control Studies

Balconi et al. (2018) carried out a randomized controlled trial to assess emotion processing in patients with schizophrenia in terms of spatial and temporal expressions in the brain using functional near-infrared spectroscopy and EEG. Their study involved 18 patients—9 in the control group and 9 who benefited from neurofeedback training. The patients were assessed before and after NF sessions using fNIRS and EEG while they were exposed to emotional images. The Self-Assessment Manikin (SAM) was applied for the subjective evaluation of the stimuli’s valence and arousal. The stimuli were represented by 100 pictures (40 positive, 40 negative, and 20 neutral) which were randomly shown on a monitor for six seconds, twelve seconds apart. The patients were required to assess the valence and the arousal, and following the experimental phase, the patients assessed their emotional experience using the SAM scale. The neurofeedback training involved a consecutive 5-week period, and the experimental group attended 10 sessions of 25 min each with a 2-minute break between sessions. EEG was recorded by placing an electrode in correspondence to the left (F3) or the right DLPFC (F4). The primary visual feedback and reward mechanism consisted of a video composed of pictures distinct from those used initially. fNIRS measured and recorded changes in the levels of oxygenated and deoxygenated hemoglobin. While it was challenging for the participants to evaluate the arousing intensity of images, all patients accurately distinguished positive, negative, and neutral stimuli before and after training, but after completing the training sessions, negative stimuli were assigned more positive values compared to the initial assessment. In both the control and experimental groups, there were higher values for the delta band in comparison to the theta, beta, and alpha bands, and the highest delta band values were encountered in frontal regions. A lateralization index was determined for each patient to evaluate frontal brain asymmetry. It was noticed that after the intervention, in the experimental group, delta activity was relatively equilibrated between the right and left hemispheres compared with the initial state, where it was predominantly right-distributed. In the control group, similar EEG findings were objected to before and after training. fNIRS results have shown that among the experimental group, the oxygenated hemoglobin levels were more equilibrated between hemispheres compared to the preintervention concentrations which were higher on the right hemisphere. The control group encountered higher oxygenated hemoglobin levels in the right hemisphere before and after their sessions [29].

Rieger et al. (2018) have investigated the therapeutic utility of neurofeedback training to modulate the auditory-evoked N100 component in patients with schizophrenia and associated auditory verbal hallucinations (AVH). Ten schizophrenia patients were selected to participate in a double-blind study, randomly assigned to the treatment group for N100 training or the control group for P200 training. Neurofeedback training involved eight double sessions over two weeks, each session lasting approximately 22 min. Only the control group showed a tendency for symptom improvement in the pre/post-comparison; however, no significant differences were found in specific hallucination-related symptoms. No significant effects of neurofeedback training were observed on the ERP components. A correlation was observed between improvement in AVH symptoms and learning across training sessions, suggesting that patients exhibiting a learning pattern characterized by within-session aptitude may benefit from EEG neurofeedback. Although neurofeedback training for the N100 component did not have a significant impact on AVH symptoms in schizophrenia patients, the identification of learning patterns and their correlation with symptom improvement provides a basis for further exploration of this approach in treating schizophrenia. The study emphasizes the importance of understanding individual learning mechanisms and their relationship with clinical outcomes in the therapeutic use of neurofeedback [30].

Balconi and Vanutelli (2019) performed a study that is a continuation of the study conducted by Balconi et al. in 2018. The objective of this randomized controlled trial was to assess the effectiveness of a neurofeedback intervention in enhancing emotional regulation among a group of patients diagnosed with schizophrenia. A total of 25 participants were involved in the study: 11 were assigned to the control group and 14 to the NF group. NF training lasted 5 weeks and consisted of 10 sessions of 25 min each with a 2 min break between sessions. There were two assessments, before and after the NF training, respectively, and both including EEG recordings, determination of hemodynamic parameters (concentration of oxygenated and deoxygenated hemoglobin) using functional near-infrared spectroscopy, and subjective evaluations regarding the valence and arousal of stimuli assessed via the Self-Assessment Manikin. One hundred images—comprising 40 positive, 40 negative, and 20 neutral—were randomly displayed to the participants on a monitor. Each image was shown for six seconds, with a twelve-second interval between them. The participants were asked to evaluate their valence, arousal, and emotional experience using the SAM scale. EEG signals were captured by positioning an electrode either on the left (F3) or the right hemisphere (F4) based on the individual electrophysiological pattern. The primary visual feedback and reward instrument consisted of a video composed of pictures distinct from those used for the baseline evaluation. Consequently, patients were tasked with improving the low-frequency range within the hemisphere displaying less activity. All patients accurately distinguished the valence of stimuli before and after training, but among the experimental group, after completing the training sessions, the negative stimuli were associated with more positive values compared to the initial assessment. Regarding fNIRS results, it was noted that oxygenated hemoglobin levels were higher for positive and negative stimuli when compared to neutral ones. Moreover, after NF training, brain activity was higher for positive and negative stimuli in the experimental group compared to the control group. There was no notable impact observed concerning arousal. In addition, after NF training, in comparison with the control group, the experimental group recorded higher levels of oxygenated hemoglobin in the right cerebral hemisphere as a reaction to negative stimuli and in the left cerebral hemisphere in response to positive stimuli [31]. Table 1 summarizes these findings.

#### 3.1.2. Non-Randomized Control Studies

F. Schneider et al. (1992) aimed to evaluate slow cortical potentials (SCP) self-regulation in schizophrenic patients in comparison to healthy controls, employing a biofeedback mechanism to visualize and train control over these potentials. Their study involved twelve schizophrenic patients and twelve control subjects, all male. Using a visual feedback system, the participants learned to control their SCPs, responding to cues that required either the increase or suppression of these potentials. Schizophrenic patients participated in twenty sessions of training, whereas controls were involved in only five, reflecting prior findings that healthy individuals can establish control within fewer sessions. The SCPs were visualized as the movement of a rocket on a screen, providing real-time feedback on participants’ control over their brain activity. Initially, schizophrenic patients showed no significant ability to differentiate or regulate their SCPs compared to controls. However, over twenty sessions, these patients developed a control level comparable to that of healthy participants by the end of the study. The final sessions demonstrated that with extensive and targeted training, even individuals with significant psychiatric conditions could learn to control their brain activity effectively. The correlation analysis revealed that greater SCP control was negatively associated with the severity of psychiatric symptoms and the frequency of hospitalization, suggesting a potential therapeutic benefit of SCP training in managing the attention-related symptoms of schizophrenia. The study concludes that schizophrenic patients can learn to self-regulate their SCPs effectively after sufficient training, similar to healthy controls, suggesting that biofeedback training could be developed as a non-invasive therapy to help mitigate some cognitive symptoms of schizophrenia [32]. Table 2 summarizes the study.

#### 3.1.3. Case Series

Schneider and Pope (1982) carried out a study to verify if EEG feedback techniques applied to a group of schizophrenic patients might have a similar effect to treatment with antipsychotics. The assumption was that feedback techniques could lead to heightened power in the distinctive alpha frequencies, while simultaneously reducing power in the characteristic slow and fast frequencies. Nine patients with schizophrenia who were under medical treatment were included in the study, and each subject participated in five sessions, with intervals between sessions varying from 48 to 96 h. The EEG was collected from the right occipital region with reference electrodes placed on linked ears (A1 and A2). While keeping their eyes closed, the patients perceived a tone through their headphones and simultaneously a flashing light. The patients were told to try to stop the sound and light and that with practice, they would learn how to do it on their own. They demonstrated that when the sound became quieter, they were close to making both the light and sound stop. After 7 min without a visual or acoustic stimulus, the biofeedback began and lasted for 33 min. In each session, two recordings were obtained that were further analyzed by a computer program, one before and one after the biofeedback began, each lasting 7 min. Within sessions, four patients had increased alpha frequencies and decreased power in low and high frequencies, while four patients had increased alpha frequencies, diminished fast activity, and no decline in slow activity. One patient did not record any change in alpha frequency. The EEG power spectrum exhibited various changes for the entire group of subjects as power densities at 8, 9, 10, and 12 Hz showed an increase, whereas power densities at 16 Hz and across all frequencies from 27 Hz to 35 Hz exhibited a decrease. A further analysis did not show cumulative EEG modifications at the end of the study [33]. 

T. Surmeli et al. (2012) investigated the efficacy of quantitative electroencephalography (qEEG)-guided neurofeedback treatment for schizophrenia. Their study evaluated whether this method affects concurrent medical treatment and patient outcomes. Fifty-one participants, previously diagnosed with chronic schizophrenia, were included. Symptoms were assessed using the Positive and Negative Syndrome Scale (PANSS), the Minnesota Multiphasic Personality Inventory (MMPI), and the Test of Variables of Attention (TOVA). Participants underwent 60-minute NF sessions, one to two times daily, with a mean of 58.5 sessions completed within 24 to 91 days. qEEG was recorded at baseline and following treatment, guiding the NF protocol. Results showed significant clinical improvements in PANSS scores for 47 out of 48 participants who completed the treatment. Improvements were also noted in MMPI and TOVA scores for participants who could complete these assessments. The study found that 19 participants’ brain electrical activity could no longer be classified as schizophrenic after NF treatment. Additionally, 27 participants remained medication-free during the follow-up period, while others required fewer medications. This study provides evidence for the positive effects of NF in treating schizophrenia, suggesting it as a potential non-invasive therapeutic option [34].

Pazooki et al. (2019) assessed the effect that EEG neurofeedback could have on the negative symptoms of schizophrenia. Their study included one male and one female who had severe negative symptoms and good compliance with medical treatment. The intervention was divided into four phases. The first and the last phases were constrained to an initial and a one-week follow-up period assessment. The second phase was a two-week period of intervention in which the patients were vigorously instructed about the training. The participants’ sensorimotor rhythm (SMR) (12–15 HZ) was enhanced on the opposite side to the dominant hand while their theta waves (4–8 Hz) were suppressed. The third phase involved neurofeedback for over two weeks without training, with the participants being encouraged to continue the training independently using the skills they had learned. In addition to the second phase, in the third phase, beta-I (13–18 Hz) was enhanced and theta wave (4–8 Hz) was reduced at the F3 electrode. Continuous visual feedback was provided for all sessions. Participants completed 20 sessions, each lasting 35 min, with 5 min of baseline and 30 min of training. Participants were assessed using CompACT SR (computerized assessment of reaction time, alertness, and selective attention under go/no-go conditions), the Global Assessment of Functioning (GAF) Scale, and the PANSS. The results indicated notable improvements in negative symptoms that were correlated with EEG changes, including spontaneous verbal expression, enhancement of sociability, and motivation towards self-initiated activities. Both participants effectively transferred their training effects from a guided condition to an unguided one, demonstrating notable learning effects [35]. 

Amico et al. (2020) conducted a study to investigate the viability of low-resolution electromagnetic tomography analysis (LORETA) neurofeedback training with individuals suffering from treatment-resistant schizophrenia and experiencing auditory–verbal hallucinations (SZ-AVH). Four patients met the inclusion criteria, and they were under treatment with clozapine. One week before NF and one week after the last session, patients were evaluated using the Edinburgh Handedness Inventory, the Quality-of-Life Enjoyment and Satisfaction Questionnaire, the Psychotic Symptom Rating Scales, the Auditory Hallucination Rating Scale, and the Hospital Anxiety and Depression Scale. EEG was recorded at baseline and one week after the last NF session. The NF was applied once a week, and each session involved seven NF rounds of 5 min each. Full NF protocol had a duration of 20 weeks and was fulfilled by only one participant, while the others completed 11, 8, and 14 sessions, respectively. The results were equivocal, indicating that certain EEG recordings that deviated from a normal pattern at the beginning of NF showed no difference by the end, while the researchers also noticed in some cases where EEG activity that was initially abnormal did exhibit differences by the end of NF. In the patient who completed all the sessions, it was noticed that the increases in theta power were normalized, while delta power was found to be abnormally diminished at the temporal areas. In the patients who attended 11 sessions, there were no power changes identified, while theta power exhibited significant increases in the frontal, central, and temporal regions following NF in patients who completed 8 and 14 sessions, respectively [36]. 

Singh et al. (2020) carried out a study to assess the potential that EEG neurofeedback might have in enhancing working memory and cognition by teaching patients with schizophrenia to increase frontal gamma activity. Their study included 31 patients with schizophrenia or schizoaffective disorder. Participants were evaluated initially and at 4, 8, and 12 weeks during treatment, as well as 4 weeks after treatment ended, in terms of electrophysiological and behavioral aspects, while symptoms and functionality were assessed at baseline, at 12 weeks, and at 4 weeks after treatment ended. The Positive and Negative Syndrome Scale and the MATRICS Consensus Cognitive Battery (MCCB) were used. The NF involved 30 min of training twice a week for 12 weeks. Participants were instructed to engage in the n-back task while 32 channels of EEG were recorded at a sampling rate of 500 Hz. Positive reinforcement was given to encourage an augmentation in synchronized gamma band responses at the F3 and F4 electrodes. Participants were presented with a selection of video games in which success was tied to the enhancement of gamma band responses. The results indicated that the NF intervention was linked to notable enhancements in frontal gamma power, which were associated with improvements in cognition. Frontal gamma power exhibited progressive improvement during the training. The most substantial improvements across various measures were evident by week 12, indicating that the optimal treatment protocol should include 12 weeks of 12 h gamma NFB sessions. Moreover, there were significant positive changes in working memory, in speed of processing, in reasoning and problem-solving, and in psychiatric symptoms [37]. We summarize these findings in Table 3.

#### 3.1.4. Case Studies

Gary J. Schummer and Jason von Stietz (2013) conducted a study that investigated the effects of intensive neurofeedback training on a 21-year-old male diagnosed with adult-onset schizophrenia of an undifferentiated type. Over 18 months, he attended four to six sessions per week, each 30 min long. The intervention started with 66 neurofeedback sessions with electrode placement at C3 and C4 to improve electrical cortical stability, followed by 272 sessions aimed to remediate statistically significant qEEG-derived coherence impairments and an additional 192 sessions of stabilization training administered before each qEEG and when it was clinically needed. The two main variations of neurofeedback training provided to the subject during this study aimed to normalize and regulate paroxysmal brainwave activity, abnormal amplitude ratios, and other known patterns of EEG dysregulation. When the subject reached optimal ratio levels in critical frequency ranges and showed improved stability, as indicated by his maintaining lower coefficients of variation, the subject was moved to the next protocol. The second variant of neurofeedback training was the two-channel sum and, when it became available, coherence neurofeedback. It was used with cortical pairs that showed statistically significant Z-scored coherence abnormalities in the qEEG analysis. When the real-time measurement, derived after each session, indicated either a normal percentage coherence or that a point of maximum benefit had been reached, the subject was moved on to the next protocol. Following the neurofeedback training, the subject’s cognitive and emotional well-being experienced major improvements, alongside his decreased need for aripiprazole (20 mg was reduced to 7.5 mg), allowing him to return to college and graduate. After the discontinuation of neurofeedback training and at 6 months follow-up, despite pharmacological therapy augmentation, the subject was markedly psychotic, exhibiting delusions of grandeur, auditory hallucinations, and severe paranoid ideation [38]. 

W. Nan et al. (2017) investigated the effects of intensive neurofeedback training on a middle-aged (51 years) woman with chronic schizophrenia. Their study examined whether short but intensive NF could benefit schizophrenic patients who struggle with long-term training. The training focused on increasing the alpha/beta2 (20–30 Hz) ratio over four consecutive days, with a total training duration of 13.5 h. The patient showed significant changes in EEG patterns, with increased alpha amplitude and decreased beta2 amplitude, indicating successful self-regulation of brain activity. Behavioral assessments revealed improvements in short-term memory, mood, and speech patterns immediately after the training. These improvements were sustained over a 22-month follow-up period, with marked reductions in both positive and negative symptoms of schizophrenia. The patient identified visualization of natural scenery as the most effective mental strategy during the training. Despite maintaining her medication regimen throughout the study, the observed EEG and behavioral changes were attributed mainly to the NF [39].

J. N. Nestoros and N. G. Vallianatou (2022) evaluated the effects of infra-low-frequency (ILF) neurofeedback on a 38-year-old male army officer with a four-year history of schizophrenia, characterized by auditory hallucinations and delusions resistant to traditional antipsychotic medications. Their study aimed to assess the rapid impact of ILF neurofeedback on his psychotic symptoms, anxiety levels, and psychosomatic conditions. The participant underwent an initial 40-minute ILF neurofeedback session before any other intervention. Assessments using the Symptom Checklist-90 (SCL-90) Scale and the Visual Analog Scale were conducted immediately before and after the session. The results showed dramatic improvements in psychotic symptoms, anxiety, and psychosomatic conditions, even before starting psychotherapy or adjusting antipsychotic medication. This positive outcome encouraged the participant to continue with further neurofeedback and psychotherapeutic sessions. After 18 sessions, the participant reported significantly reduced and manageable levels of auditory hallucinations and delusions, particularly under stress, with no stereotypic behavior patterns. He experienced notable improvements in his general psychological state and began to engage in social activities and professional duties more effectively [40]. The main results are synthesized in Table 4.

## 4. Discussion

We review systematically the primary reports of EEG-NF as a therapeutical tool for schizophrenia. These findings highlight the potential of NF as a personalized therapeutic intervention for schizophrenia, offering significant benefits that complement traditional pharmacological and psychotherapeutic treatments, revealing promising results in patient outcomes across various domains, including cognitive function, emotional regulation, and symptom management.

Cognitive improvements have been observed by Singh et al. (2020), who demonstrated that NF increases frontal gamma activity, resulting in significant cognitive and symptom improvements, such as in working memory, processing speed, and reasoning abilities, with the best results achieved after a 12-week protocol [37]. These results align with the existing literature suggesting that NF can enhance cognitive performance by modulating brainwave activity, particularly in the gamma frequency band [41,42]. Furthermore, Surmeli et al. (2012) have used the Z score qEEG protocol, reporting significant clinical improvements in cognitive functions as measured via PANSS scores and other assessments [34]. Pazooki et al. (2019) also showed that NF targeting sensorimotor rhythm (SMR) and beta-I waves could significantly improve negative symptoms and cognitive functions [35]. 

The study of Schummer and von Stietz (2013) demonstrated substantial cognitive and emotional improvements in a young adult male with schizophrenia following 530 NF sessions over 18 months, allowing for a reduction in medication and a successful return to academic life, although benefits waned after discontinuation [38]. Nan et al. (2017) showed that a middle-aged woman with chronic schizophrenia could achieve sustained improvements in EEG patterns, memory, mood, and speech after a short but intensive period of NF training, with benefits lasting for 22 months [39]. 

Emotional regulation and symptom management have been documented in the following studies. Balconi el al. (2018) used fNIRS and EEG to modulate emotional processing, showing that NF led to more balanced hemispheric activity and more positive evaluations of negative stimuli. Moreover, delta band activity and oxygenated hemoglobin levels became more equilibrated between hemispheres in the experimental group after training, suggesting improved emotional regulation and neural plasticity [29]. 

Building on their 2018 study, Balconi and Vanutelli (2019) further demonstrated the effectiveness of NF training in enhancing emotional regulation, confirming their initial findings. They observed that after NF training, patients in the experimental group assigned more positive values to negative stimuli and showed increased brain activity for both positive and negative stimuli as measured by higher oxygenated hemoglobin levels in response to these stimuli compared to the control group. This further supports the role of NF training in modulating emotional responses and enhancing neurophysiological balance [31]. Rieger et al. (2018) investigated NF training targeting the auditory-evoked N100 component in schizophrenia patients with auditory verbal hallucinations (AVH). Although the study found no significant effects on ERP components or specific AVH symptoms, it highlighted the correlation between symptom improvement and learning patterns across sessions, suggesting individual differences in response to NF training [30]. 

In the single non-randomized controlled study, F. Schneider et al. (1992) found a negative correlation between the degree of SCP control and the severity of psychiatric symptoms and hospitalization frequency, indicating that enhanced SCP self-regulation could potentially reduce symptom severity and improve clinical outcomes [32].

Amico et al. (2020) had mixed results with LORETA NF, showing some EEG normalization but inconsistent outcomes [36]. Singh et al. (2020) demonstrated significant symptom improvements associated with increased frontal gamma activity [37]. 

Nestoros and Vallianatou (2022) reported rapid and significant reductions in psychotic symptoms and anxiety in an army officer with schizophrenia after just one ILF NF session, with continued improvements following further sessions [40]. Schummer and von Stietz (2013) and Nan et al. (2017) also reported notable emotional improvements, with patients showing reduced symptom severity and improved mood [38,39]. 

The potential medication dependency reduction has been observed in one case series and one case report, as follows.

Surmeli et al. (2012) found that many participants could reduce or eliminate their medication use following effective NF training. This suggests that NF can serve as a complementary intervention, reducing reliance on pharmacological treatments [34].

Schummer and von Stietz (2013) demonstrated a significant reduction in medication dosage for a young adult male with schizophrenia, though the benefits waned after discontinuation of NF training [38].

The clinical trials included present several limitations that need to be addressed.

One major limitation is the lack of randomization and proper blinding methods in many of the studies, which can introduce biases and limit the generalizability of the findings. Additionally, the absence of comparisons to gold-standard treatments like medication therapy weakens the evidence base. Without direct comparisons, it is challenging to determine whether NF offers superior or complementary benefits compared to existing treatments.

The sample size of the studies is small; none of the studies has at least a total of 50 patients, with the experimental group being even smaller, thus affecting the reliability of the results. This leads to statistical underpowering, making it difficult to detect significant effects and reducing the reliability of the results.

A critical methodological limitation is the challenge of conclusively attributing improvements in functioning to changes in brain activity based on typical NF methodologies. The current methodologies often cannot distinguish between the direct effects of NF on brain activity and other factors such as participant expectations and placebo effects.

Therefore, this leaves a huge gap that needs to be filled, and to do so, researchers should implement a better protocol design with a larger population [43] and include placebo arms. 

Placebo can have a significant influence on interventional studies. Moreover, NF relies on non-specific mechanisms of action that can lead to a very specific physiological change; thus, the overall effect may stem more from placebo-like effects rather than the intervention itself. To overcome this, the scientist must define clear and quantifiable outcomes and provide sham feedback in the control arm to differentiate the genuine effects of the intervention from other non-specific influences involving expectation and motivation [44].

Skalski (2020), in an RCT study on children with ADHD, observed that children from the intervention group who were not informed about the placebo possibility inclusion arm performed better than children in the placebo-informed arm. The expectation of a placebo can diminish the effectiveness of the intervention by undermining individuals’ sense of agency and moral attitudes, leading to greater external attribution and reduced efforts and outcomes [45]. 

Furthermore, the heterogeneity in NF protocols, target frequencies, and training durations across studies complicates the synthesis of findings and the formulation of standardized treatment guidelines. The variability in protocols, such as the different brainwave targets (e.g., gamma, alpha, SMR) and electrode placements, suggests that individualized approaches may be necessary; it also highlights the need for more systematic research to identify the most effective strategies.

The strengths of the reviewed studies lie in their innovative approaches and the potential for long-lasting benefits as demonstrated by some case reports suggesting that NF can result in substantial clinical improvements in cognitive functions and negative symptoms. These studies contribute valuable insights into the potential mechanisms by which NF might exert its therapeutic effects, such as improved neural plasticity and emotional regulation. Moreover, the longitudinal benefits observed in some case studies indicate that NF could offer sustained symptom relief and functional gains. These findings suggest that NF might not only complement existing treatments but also provide a non-invasive alternative that empowers patients to self-regulate their brain activity.

Overall, the studies highlight the potential use of NF to influence neuronal activity in schizophrenia patients, despite heterogeneous degrees of symptom improvement. The findings emphasize the importance of individualized approaches to NF training, considering the observed variability in learning patterns and clinical outcomes. 

In recent years, a growing interest in neuromodulation has been seen, mainly as a consequence of computer and engineering science that has made extraordinary computational advances. 

Neuromodulation shapes emotional learning in various circuits that are disturbed across the brain. The net result of its activity is to change downstream neurons from the baseline state, thus changing their mode of encoding [46]. This concept refers mainly to a direct brain-based technique with the aim of targeting psychiatric disorders and alleviating dysfunction. These techniques differ from traditional therapies by not aiming to treat syndromes but rather various patterns of behaviors arising from neuronal network impairments [18]. 

The various wave modulation protocols (alpha, beta, alpha/beta, delta, gamma, and theta), multiple electrode placements (dependent on the area of interest), and types of NF (EEG, fMRI, etc.) [47] can assure a personalized case-based approach for heterogenic disorders that are complex and variable in their symptoms. 

Although the principle of NF is uniform through the studies included in this paper, the protocols used for schizophrenia have a wide variability. The consensus on what protocol to use is hard to reach mainly because there is no single answer to the central pathophysiology mechanism behind schizophrenia, and no neurodiagnostic or humoral markers are defined [48,49]. 

From the included studies, neurofeedback training seems to modify neuronal activity and the metabolism of the brain; the findings are sustained by changes measured via neuroimaging techniques and electrical activities measured on the scalp pre- and post-intervention. More still, these changes are sustained even when training is not provided, i.e., between sessions. The studies with follow-up periods suggest that the changes are generalized, being independent of NF sessions. 

Continued research is essential to optimize NF protocols and understand the mechanisms underlying their therapeutic effects, aiming to enhance emotional regulation and reduce symptom severity in schizophrenia. 

Another aspect drawn from this review is that the development of specific NF training for a specific disorder is premature. This is due to the weak evidence regarding the associations between training in a specific frequency band and the performance measurements upon which the intervention relies. Despite the large body of publications associated with neurofeedback terms, there is a scarcity of well-designed randomized control studies in the literature, the balance being inclined toward case reports/series. 

It is important to choose sustainable target regions and to identify the treatment dose response. In the lesional studies, it was observed that specific regions of the brain produce specific symptoms mostly related to the region. For example, the frontal lobes are responsible for immediate and sustained attention, time management, social skills, emotions, empathy, working memory, and executive planning. Each region represents a specific feeling or task; thus, identification of these areas orientates the neurofeedback treatment [48].

Other questions that need to be addressed are how long a session should last and how it should be spaced over time. It is argued that a training session should not be too long or too short, thus not exhausting the patient. Alternatively, the changes require a minimal amount of time; it seems that 20–30 min is the sweet spot [50,51]. Regarding time spacing, no consensus has yet been reached, but similar to another type of learning, it was observed that spacing training over a period of days or weeks can be more efficient than mass training in a single day [52]. 

This intervention has the potential to empower schizophrenic patients with the tools to improve their brain function, therefore increasing everyday functionality and resulting in significant individual and social benefits.

## 5. Conclusions

This body of research suggests the potential use of neurofeedback as an intervention with a therapeutical role in schizophrenia, highlighting its ability to improve cognitive function, emotional regulation, and overall symptom management. 

Further research is warranted to better understand the mechanisms driving these improvements, therapy outcomes, and the variables that influence it and optimize NF protocols.

Future studies need to investigate and establish the optimal training parameters, such as the time of a session, the time between sessions, and the optimal modulation site, as well as the combination of NF with other drugs and techniques that can alleviate the symptoms of the disorder.

## Figures and Tables

**Table 1:** Randomized controlled studies.

Reference	Design 1. Protocol 2. Randomized (Y/N) 3. Blinded 4. Number of Sessions 5. Duration of a Session 6. Follow Up	Sample 1. N (Sex) 2. Age 3. Medication (Y/N)	Measures Used	Results
M. Balconi et al. (2018) [29]	1. EEG NF Reward 0.5–5.5 Hz; inhibit—low = 0.1–0.5 Hz; inhibit—high = 50–64 Hz 2. Y 3. Single 4. 10 5. 25 min 6. No	1. 18 (9 M; 9 F) 2. 34.12 ± 7.09 3. YES	EEG; SAM.	More functional management of negative affects after NF. NF could restore a balanced interhemispheric state measured via oxygenated hemoglobin levels.
K. Rieger et al. (2018) [30]	1. N100 ERP modulation NF 2. Y 3. Double 4. 16 5. 22 min 6. No	1. 10 (6 M; 4 F) 2. 36.6 ± 14.6 3. YES	EEG; PANSS; PsyRat; HVLR-R; NAB-Mazes; D2 test of attention; HCS.	No significant pre/post intervention differences in AVH symptoms assed with HCS.A positive association between learning factors and improvements in hallucinations. No relation between psychotic symptoms and NF performance. No significant pre/post intervention spatial difference or GFP effect on N100. A significant spatial pre/post difference for P200(control group) with a tendency for higher GFP post-training.
Balconi and Vanutelli (2019) [31]	1. EEG NF reward = 0.5–5.5 Hz; inhibit—low = 0.1–0.5 Hz; inhibit—high = 50–64 Hz (F3, F4) 2. Y 3. Single 4. 10 5. 25 min 6. No	1. 25 (13 M; 12 F) 2. 32.10 ± 4.76	fNIRS; SAM.	After the intervention, the perception of negative and positive stimuli received a more positive value compared to the initial assessment. Increase in oxygenated hemoglobin over frontopolar regions for positive and negative stimuli compared to neutral ones.

EEG—electroencephalogram; fNIRS—functional near-infrared spectroscopy; HCS—Hallucination Change Scale; HVLR—R-Hopkins Verbal Learning Test (Revised); NAB-Mazes—Neuropsychological Assessment; MCCB—MATRIC Consensus Cognitive Battery; PANSS—Positive and Negative Syndrome Scale; PsyRat—Psychotic Rating Scale; SAM—Self-Assessment Manikin.

**Table 2:** Non-randomized control studies.

Reference	Design 1. Protocol 2. Randomized (Y/N) 3. Blinded 4. Number of Sessions 5. Duration of a Session 6. Follow Up	Sample 1. N (Sex) 2. Age 3. Medication (Y/N)	Measures Used	Results
F. Schneider et al. (1992) [32]	1. Regulation of activity of SCP (Cz) 2. N 3. N 4. 20 5. 20 min 6. NO	1.12 M SCH vs. 12 HP 2. 27.3 ± 3.93 vs. 27.5 ± 4.2	EEG	Patients need more NF training compared to the control group to achieve the same slow cortical potential control.

EEG—electroencephalogram; HP—healthy patients; SCH—patients with schizophrenia; SCP—slow cortical potentials.

**Table 3:** Case series studies.

Reference	Design 1. Protocol 2. Randomized (Y/N) 3. Blinded 4. Number of Sessions 5. Duration of a Session 6. Follow Up	Sample 1. N (Sex) 2. Age 3. Medication (Y/N)	Measures Used	Results
S. J. Schneider and A. T. Pope (1982) [33]	1. Increase in 8–13 Hz waves and decrease in power densities over 15 Hz (O2) 2. N 3. N 4. 5 5. 33 min 6. No	1. 9 (4 M; 1 F) 2. 45.4 ± 14.1 3. Y	EEG.	Within the session, there was an increase in power densities between 8–13 Hz and a decrease in power densities over 15 Hz. There were no significant changes between sessions.
T. Surmeli et al. (2012) [34]	1. Z score qEEG NF 2. NO 3. NO 4. 58.5 (24–91) 5. 60 min 6. 1 month to 1 year	1. 51 (25 M; 26 F) 2. 28.8 ± 7.9 3. Y (7hl wash out)	qEEG; PANSS; MMPI; TOVA.	A significant improvement in PANSS with a 20% or more score decrease. A trend to normalization in all the MMPI. Normalization in the visual subset of TOVA; only omission errors and reaction time variability showed statistically significant improvement. Brain electrical activity changes: 19 patients have normalized it to typical levels, with a 73% increase in alpha wave and a 20% increase in theta wave; hyper-coherence was seen in 63% of the cases, with a decrease to 58% at the end of the study.
K. Pazooki et al. (2019) [35]	1. SMR augmentation and theta inhibition opposed to the handiness 2. N 3. NO 4. 20 5. 35 min 6. one week	1. 2 (1 M; 1 F) 2. 30 and 45 3. Y	EEG; CompACT-SR; GAFS; PANSS.	Significant learning effect concerning EEG parameters. Significant improvement of negative symptomatology and a marked increase in spontaneous verbal behavior and sociability.
F. Amico et al. (2020) [36]	1. LORETA Z-score 2. N 3. No 4. 8–14 5. 45 min 6. No	1. 4 (?) 2. Between 30–59 3. ?	EEG.	Heterogenous results: two patients showed widespread non-normative theta power while the other two did not.
F. Singh et al. (2020) [37]	1. Gamma band NF vs. placebo (F3, F4) 2. Y 3. Double 4. 24 5. 30 min 6. Week 14	1. 31 (15 M; 16 F) vs. 12(?) 2. 45.4 ± 9.55 vs. ? 3. YES	WM by n-back task (32 channels qEEG); MCCB; PANSS.	Significant improvement on the 1-back task at 2, 4, and 12 weeks; significant changes in the 2-back task at week 12. Significant effect on MCCB total score at weeks 4 and 8, with the largest effect at week 12. Significant improvements in working memory, reasoning and problem solving, and speed of processing, with a trend for visual learning. Improvements in PANSS positive, negative, and total scores. Follow-up effects at week 16 compared to baseline: greater FGP during 2-back test; marginally better 2-back performance; greater MCCB score PANSS total, significantly lower positive and negative symptoms.

CompACT-SR—computerized assessment of reaction time, alertness, and selective attention under go/no-go conditions; EEG—electroencephalogram; FGP—frontal gamma power; GAFS—Global Assessment of Functioning Scale; MCCB—the MATRICS Consensus Cognitive Battery; MMPI—Minnesota Multiphasic Personality Inventory; ?—no data.

**Table 4:** Case studies.

Reference	Design 1. Protocol 2. Randomized (Y/N) 3. Blinded 4. Number of Sessions 5. Duration of a Session 6. Follow Up	Sample 1. N (Sex) 2. Age 3. Medication (Y/N)	Measures Used	Results
G. J. Schummer and J. von Stietz (2013) [38]	1. Normalization of abnormal amplitude ration and patterns of EEG dysregulation (C3 and C4) 2. N 3. N 4. 530 5. 30 min 6. NO	1. 1 M 2. 21 3. Y	EEG	Major cognitive and emotional improvements. Significant reduction in symptoms and a reduction in the needed for the efficient dose of antipsychotic (from 20 mg to 7.5 mg). Relapse of the symptoms after 6 months of stopping NF and continuing medication (with the necessary dose increased).
W. Nan et al. (2017) [39]	1. Increase alpha and beta2 decrease (P4) 2. N 3. - 4. 4 5. total 12.5 h 6. 22 months	1. 1 (f) 2. 51 3. Y	EEG; Short-Term Memory Test	Increased trend in the alpha band and a decrease in the beta2 band. Improvement in short memory test. Dramatic improvement in positive and negative symptoms after 22 months follow-up.
J. N. Nestoros and N. G. Vallianatou (2022) [40]	1. Infra-Low Frequency NF/10 2. N 3. NO 4. 18 5. 60 min 6. NO	1. 1 (M) 2. 38 3. YES	EEG; SCL-90; Visual Analog Scale	Higher and predominantly delta band activity in all recorded areas; ILF/10 was significantly lower than delta. After the first session (SCL-90), there were lower levels of somatization, obsessive–compulsive symptoms, anxiety, paranoid ideation, and psychoticism. At the final session (SCL-90), there was a significant positive result in the mean scores of anxiety, paranoid ideation, and psychoticism after the final session. Visual analog scale: after the first session, an improvement in anxiety, psychotically state, psychosomatic symptoms, auditory hallucination, and delusional thinking was observed. After the final session, significant positive changes in auditory hallucination, psychotically state, anxiety, and delusional thinking were observed.

EEG—electroencephalogram; SCL-90—The Symptom Checklist-90.

## Data Availability

No new data were created or analyzed in this study. Data sharing is not applicable to this article.

## References

[1] WHO Schizophrenia. https://www.who.int/news-room/fact-sheets/detail/schizophrenia?gad_source=1&gclid=CjwKCAjw8diwBhAbEiwA7i_sJdFvvOSoic6Fwi-QY7IvqsAj8hPnqxVT_i4_pQG_SnVkU-hv0BR1WRoCSkkQAvD_BwE.

[2] Marder S.R., Cannon T.D. (2019). Schizophrenia. N. Engl. J. Med..

[3] World Health Organization (2004). ICD-10: International Statistical Classification of Diseases and Related Health Problems: Tenth Revision.

[4] Velligan D.I., Rao S. (2023). The epidemiology and global burden of schizophrenia. J. Clin. Psychiatry.

[5] Amoretti S., Mezquida G., Verdolini N., Bioque M., Sánchez-Torres A.M., Pina-Camacho L., Zorrilla I., Trabsa A., Rodriguez-Jimenez R., Corripio I. (2023). Negative symptoms and sex differences in first episode schizophrenia: What’s their role in the functional outcome? A longitudinal study. Span. J. Psychiatry Ment. Health.

[6] Li R., Ma X., Wang G., Yang J., Wang C. (2016). Why sex differences in schizophrenia?. J. Transl. Neurosci..

[7] Lamsma J., Raine A., Kia S.M., Cahn W., Arold D., Banaj N., Barone A., Brosch K., Brouwer R., Brunetti A. (2024). Structural brain abnormalities and aggressive behaviour in schizophrenia: Mega-analysis of data from 2095 patients and 2861 healthy controls via the ENIGMA consortium. medRxiv.

[8] Walton E., Hibar D.P., Van Erp T.G., Potkin S.G., Roiz-Santiañez R., Crespo-Facorro B., Suarez-Pinilla P., van Haren N.E., De Zwarte S., Kahn R.S. (2018). Prefrontal cortical thinning links to negative symptoms in schizophrenia via the ENIGMA consortium. Psychol. Med..

[9] Goghari V.M., Sponheim S.R., MacDonald III A.W. (2010). The functional neuroanatomy of symptom dimensions in schizophrenia: A qualitative and quantitative review of a persistent question. Neurosci. Biobehav. Rev..

[10] Firth J., Cotter J., Carney R., Yung A.R. (2017). The pro-cognitive mechanisms of physical exercise in people with schizophrenia. Br. J. Pharmacol..

[11] Cattarinussi G., Di Giorgio A., Sambataro F. (2024). Cerebellar dysconnectivity in schizophrenia and bipolar disorder is associated with cognitive and clinical variables. Schizophr. Res..

[12] Tuppurainen H., Määttä S., Könönen M., Julkunen P., Kautiainen H., Hyvärinen S., Vaurio O., Joensuu M., Vanhanen M., Aho-Mustonen K. (2024). Navigated and individual α-peak-frequency-guided transcranial magnetic stimulation in male patients with treatment-refractory schizophrenia. J. Psychiatry Neurosci..

[13] Ribeiro T.F., Carriello M.A., de Paula E.P., Garcia A.C., Rocha G.L.d., Teive H.A.G. (2023). Clinical applications of neurofeedback based on sensorimotor rhythm: A systematic review and meta-analysis. Front. Neurosci..

[14] Legarda S.B., McMahon D., Othmer S., Othmer S. (2011). Clinical neurofeedback: Case studies, proposed mechanism, and implications for pediatric neurology practice. J. Child Neurol..

[15] Dinh S.T., Nickel M.M., Tiemann L., May E.S., Heitmann H., Hohn V.D., Edenharter G., Utpadel-Fischler D., Tölle T.R., Sauseng P. (2019). Brain dysfunction in chronic pain patients assessed by resting-state electroencephalography. Pain.

[16] Hammond D.C. (2011). What is neurofeedback: An update. J. Neurother..

[17] Thibault R.T., Lifshitz M., Raz A. (2016). The self-regulating brain and neurofeedback: Experimental science and clinical promise. Cortex.

[18] Gandara V., Pineda J.A., Shu I.-W., Singh F. (2020). A systematic review of the potential use of neurofeedback in patients with schizophrenia. Schizophr. Bull. Open.

[19] Kohl S.H., Mehler D.M.A., Lührs M., Thibault R.T., Konrad K., Sorger B. (2020). The Potential of Functional Near-Infrared Spectroscopy-Based Neurofeedback—A Systematic Review and Recommendations for Best Practice. Front. Neurosci..

[20] Buch E.R., Modir Shanechi A., Fourkas A.D., Weber C., Birbaumer N., Cohen L.G. (2012). Parietofrontal integrity determines neural modulation associated with grasping imagery after stroke. Brain.

[21] Patil A.U., Lin C., Lee S.H., Huang H.W., Wu S.C., Madathil D., Huang C.M. (2023). Review of EEG-based neurofeedback as a therapeutic intervention to treat depression. Psychiatry Res. Neuroimaging.

[22] Tolin D.F., Davies C.D., Moskow D.M., Hofmann S.G. (2020). Biofeedback and Neurofeedback for Anxiety Disorders: A Quantitative and Qualitative Systematic Review. Adv. Exp. Med. Biol..

[23] Butler L.K., Kiran S., Tager-Flusberg H. (2020). Functional Near-Infrared Spectroscopy in the Study of Speech and Language Impairment Across the Life Span: A Systematic Review. Am. J. Speech Lang. Pathol..

[24] Kim J.W., Jeong H., Choi T.Y., Moon J.Y. (2023). 2.60 The Effect of Mobile Neurofeedback on Internet Addiction in Neurotypical Children: A Double-Blind, Sham-Controlled RCT. J. Am. Acad. Child Adolesc. Psychiatry.

[25] Saif M.G.M., Sushkova L. (2023). Clinical efficacy of neurofeedback protocols in treatment of Attention Deficit/Hyperactivity Disorder (ADHD): A systematic review. Psychiatry Res. Neuroimaging.

[26] Roy R., de la Vega R., Jensen M.P., Miró J. (2020). Neurofeedback for Pain Management: A Systematic Review. Front. Neurosci..

[27] Imperatori C., Mancini M., Della Marca G., Valenti E.M., Farina B. (2018). Feedback-Based Treatments for Eating Disorders and Related Symptoms: A Systematic Review of the Literature. Nutrients.

[28] Moher D., Shamseer L., Clarke M., Ghersi D., Liberati A., Petticrew M., Shekelle P., Stewart L.A., Group P.-P. (2015). Preferred reporting items for systematic review and meta-analysis protocols (PRISMA-P) 2015 statement. Syst. Rev..

[29] Balconi M., Frezza A., Vanutelli M.E. (2018). Emotion Regulation in Schizophrenia: A Pilot Clinical Intervention as Assessed by EEG and Optical Imaging (Functional Near-Infrared Spectroscopy). Front. Hum. Neurosci..

[30] Rieger K., Rarra M.H., Diaz Hernandez L., Hubl D., Koenig T. (2018). Neurofeedback-Based Enhancement of Single-Trial Auditory Evoked Potentials: Treatment of Auditory Verbal Hallucinations in Schizophrenia. Clin. EEG Neurosci..

[31] Balconi M., Vanutelli M.E. (2019). Neurofeedback intervention for emotional behavior regulation in schizophrenia: New experimental evidences from optical imaging. NeuroRegulation.

[32] Schneider F., Rockstroh B., Heimann H., Lutzenberger W., Mattes R., Elbert T., Birbaumer N., Bartels M. (1992). Self-regulation of slow cortical potentials in psychiatric patients: Schizophrenia. Biofeedback Self Regul..

[33] Schneider S.J., Pope A.T. (1982). Neuroleptic-like electroencephalographic changes in schizophrenics through biofeedback. Biofeedback Self-Regul..

[34] Surmeli T., Ertem A., Eralp E., Kos I.H. (2012). Schizophrenia and the efficacy of qEEG-guided neurofeedback treatment: A clinical case series. Clin. EEG Neurosci..

[35] Pazooki K., Leibetseder M., Renner W., Gougleris G., Kapsali E. (2019). Neurofeedback Treatment of Negative Symptoms in Schizophrenia: Two Case Reports. Appl. Psychophysiol. Biofeedback.

[36] Amico F., Keane M., Lee M., McCarthy-Jones S. (2020). A Feasibility Study of LORETA Z-Score Neurofeedback Training in Adults with Schizophrenia-Spectrum Disorder Experiencing Treatment-Resistant Auditory Verbal Hallucinations. NeuroRegulation.

[37] Singh F., Shu I.W., Hsu S.H., Link P., Pineda J.A., Granholm E. (2020). Modulation of frontal gamma oscillations improves working memory in schizophrenia. NeuroImage Clin..

[38] Schummer G.J., von Stietz J. (2013). QEEG Guided Neurofeedback to Treat Schizophrenia: A Case Study. J. Neurother..

[39] Nan W., Wan F., Chang L., Pun S.H., Vai M.I., Rosa A. (2017). An Exploratory Study of Intensive Neurofeedback Training for Schizophrenia. Behav. Neurol..

[40] Nestoros J.N., Vallianatou N.G. (2022). Infra-Low Frequency Neurofeedback rapidly ameliorates schizophrenia symptoms: A case report of the first session. Front. Hum. Neurosci..

[41] Shu I.W., Granholm E.L., Singh F. (2023). Targeting Frontal Gamma Activity with Neurofeedback to Improve Working Memory in Schizophrenia. Curr. Top. Behav. Neurosci..

[42] Andrade K., Houmani N., Guieysse T., Razafimahatratra S., Klarsfeld A., Dreyfus G., Dubois B., Vialatte F., Medani T. (2024). Self-Modulation of Gamma-Band Synchronization through EEG-Neurofeedback Training in the Elderly. J. Integr. Neurosci..

[43] Schweizer G., Furley P. (2016). Reproducible research in sport and exercise psychology: The role of sample sizes. Psychol. Sport Exerc..

[44] Thibault R.T., Lifshitz M., Raz A. (2017). Neurofeedback or neuroplacebo?. Brain.

[45] Skalski S. (2022). Impact of placebo-related instruction on HEG biofeedback outcomes in children with ADHD. Appl. Neuropsychol. Child.

[46] Likhtik E., Johansen J.P. (2019). Neuromodulation in circuits of aversive emotional learning. Nat. Neurosci..

[47] Marzbani H., Marateb H.R., Mansourian M. (2016). Methodological Note: Neurofeedback: A Comprehensive Review on System Design, Methodology and Clinical Applications. Basic Clin. Neurosci. J..

[48] Warren N., O′Gorman C., Horgan I., Weeratunga M., Halstead S., Moussiopoulou J., Campana M., Yakimov V., Wagner E., Siskind D. (2024). Inflammatory cerebrospinal fluid markers in schizophrenia spectrum disorders: A systematic review and meta-analysis of 69 studies with 5710 participants. Schizophr. Res..

[49] Rowbal T., Kajy M., Burghardt K.J. (2023). Epigenome-wide studies of antipsychotics: A systematic review and pathway meta-analysis. Epigenomics.

[50] Ghaziri J., Tucholka A., Larue V., Blanchette-Sylvestre M., Reyburn G., Gilbert G., Lévesque J., Beauregard M. (2013). Neurofeedback Training Induces Changes in White and Gray Matter. Clin. EEG Neurosci..

[51] Mirifar A., Beckmann J., Ehrlenspiel F. (2017). Neurofeedback as supplementary training for optimizing athletes’ performance: A systematic review with implications for future research. Neurosci. Biobehav. Rev..

[52] Vernon D., Frick A., Gruzelier J. (2004). Neurofeedback as a treatment for ADHD: A methodological review with implications for future research. J. Neurother..

